# Supplementary material for: Single‐cell analyses reveal SARS‐CoV‐2 interference with intrinsic immune response in the human gut
Source: Mol Syst Biol. 2021 Apr 27;17(4):e10232. doi: 10.15252/msb.202110232 (PMC8077299; doi:10.15252/msb.202110232)
Supplement: Supplementary file 1 — Appendix [file MSB-17-e10232-s001.docx]

**Table of contents**

**Appendix Figure S1:** General information of single-cell RNA-seq

**Appendix Figure S2:** Identification of SARS-CoV-2 infected cells using SoupX on targeted scRNAseq datasets

**Appendix Figure S3:** Gene expression profiles showing the top 30 genes with highest fold change in the differential expression analysis

**Appendix Figure S4:** Gene enrichment analysis in infected and bystander cells upon SARS-CoV-2 infection

**Appendix Figure S5:** Cell type specific differential response of infected *vs.* bystander cells upon SARS-CoV-2 infection of colon-derived organoids

**Appendix Figure S6:** Cell type specific differential response of infected vs. bystander cells upon SARS-CoV-2 infection of colon-derived organoids

**Appendix Figure S7:** Cell type specific differential response of infected *vs.* bystander cells upon SARS-CoV-2 infection of ileum-derived organoids

**Appendix Figure S8:** Cell type specific differential response of infected vs. bystander cells upon SARS-CoV-2 infection of ileum-derived organoids

**Appendix Figure S9:** Interferon induction requires virus replication

**
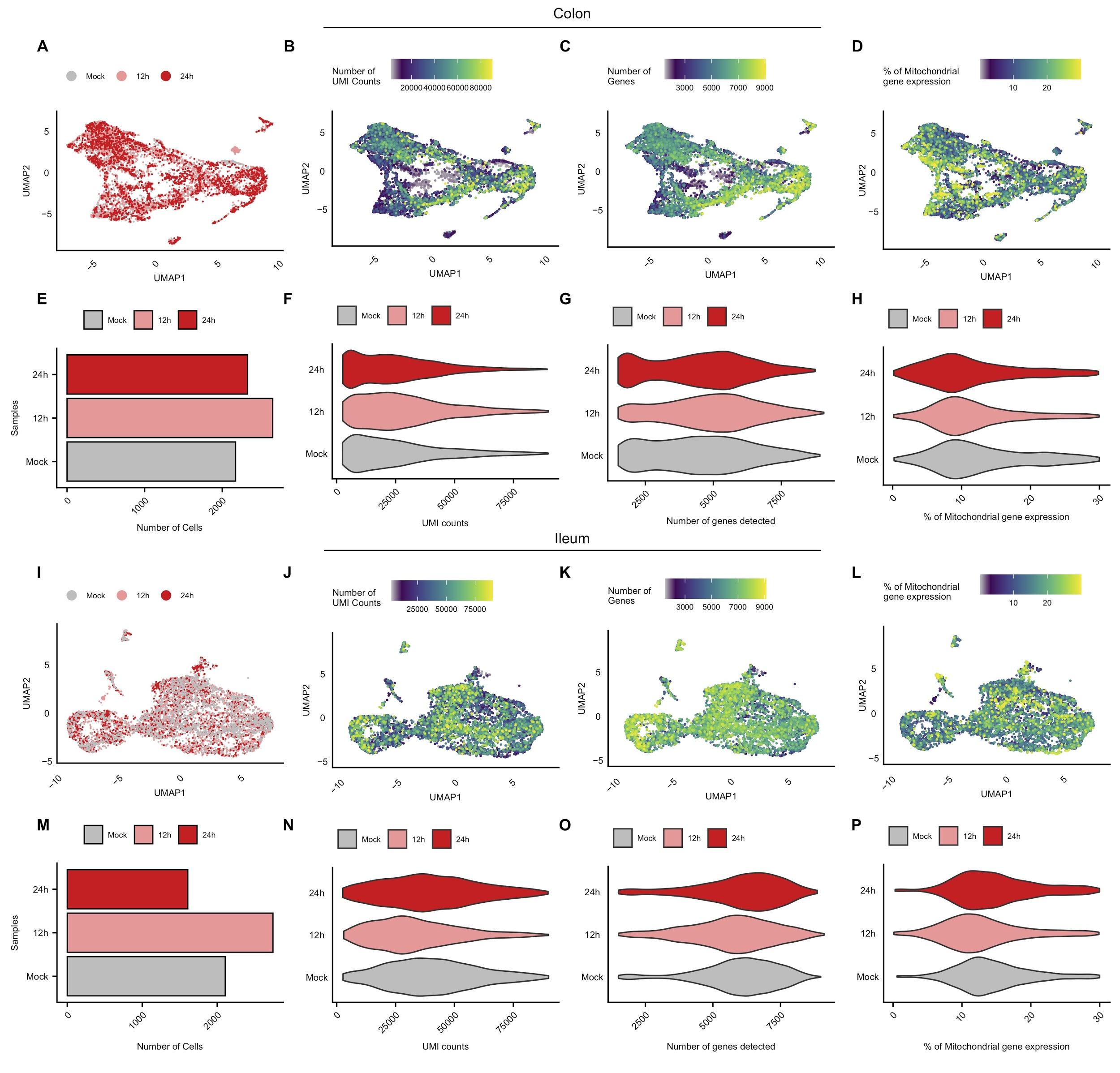
**

**Appendix Figure S1. General information of single-cell RNA-seq. A-H**. Data correspond to scRNAseq of mock and SARS-CoV-2 infected colon organoids. **I-P**. Data correspond to scRNAseq of mock and SARS-CoV-2 infected ileum organoids. **A.** and **I.** Uniform manifold approximation and projection (UMAP) embedding of scRNA-Seq data from mock and SARS-CoV-2 infected organoids at 12 and 24 hpi. Conditions are color coded. **B.** and **J.** Number of UMI counts. **C.** and **K.** Number of genes sequenced in each cell, **D.** and **L.** Percentage of mitochondrial counts **E.** and **M.** Number of cells in scRNAseq datasets for each condition (mock, 12 and 24 hpi). **F.** and**.** Violin plots depicting the number of UMI per cell for each condition (mock, 12 and 24 hpi). **G.** and **O.** Violin plots depicting the number of genes detected per cell for each condition (mock, 12 and 24 hpi). **H.** and **P.** Violin plots depicting the percentage of mitochondrial counts for each condition (mock, 12 and 24 hpi).

**
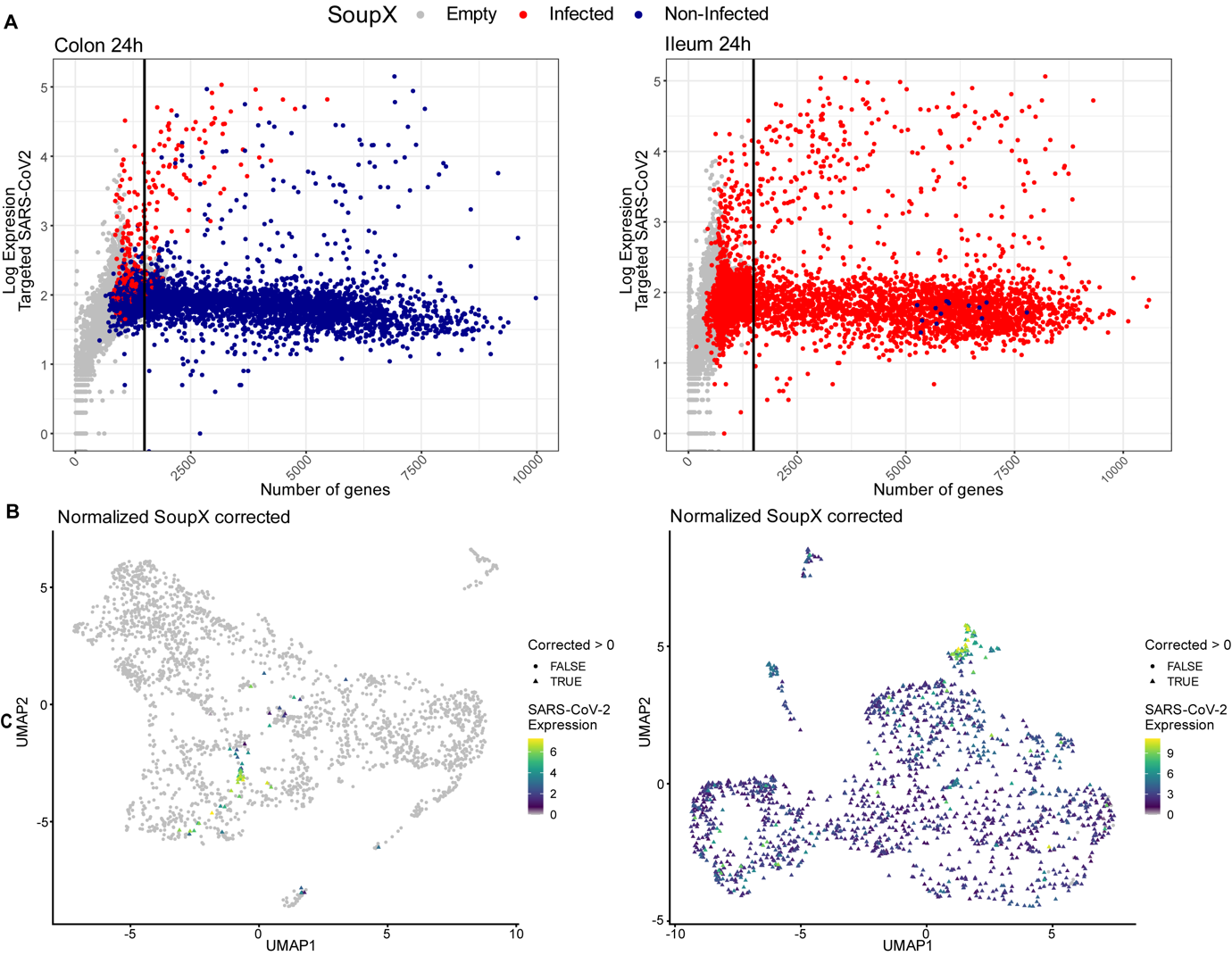
**

**Appendix Figure S2. Identification of SARS-CoV-2 infected cells using SoupX on targeted scRNAseq** **datasets.** **A.** SARS-CoV-2 expression as function of the number of genes per droplet from the targeted scRNAseq datasets of colon and Ileum organoids at 24 hpi. Droplet types are colored for droplets containing infected and non infected cells after SoupX correction and empty droplets. **B.**  Uniform manifold approximation and projection (UMAP) embedding of the scRNA-Seq data of infected colon organoids depicting SARS-CoV-2 infected cells.

**Appendix Figure S3. Gene expression profiles showing the top 30 genes with highest fold change in the differential expression analysis. A.** Gene expression profiles of immature enterocytes 2 subpopulation in colon-derived organoids 24 hpi**.** (Left) Infected *vs*. mock infected cells at 24 hpi and (right) bystander *vs.* mock infected cells. **B.** Same as A but for ileum-derived organoids.

**
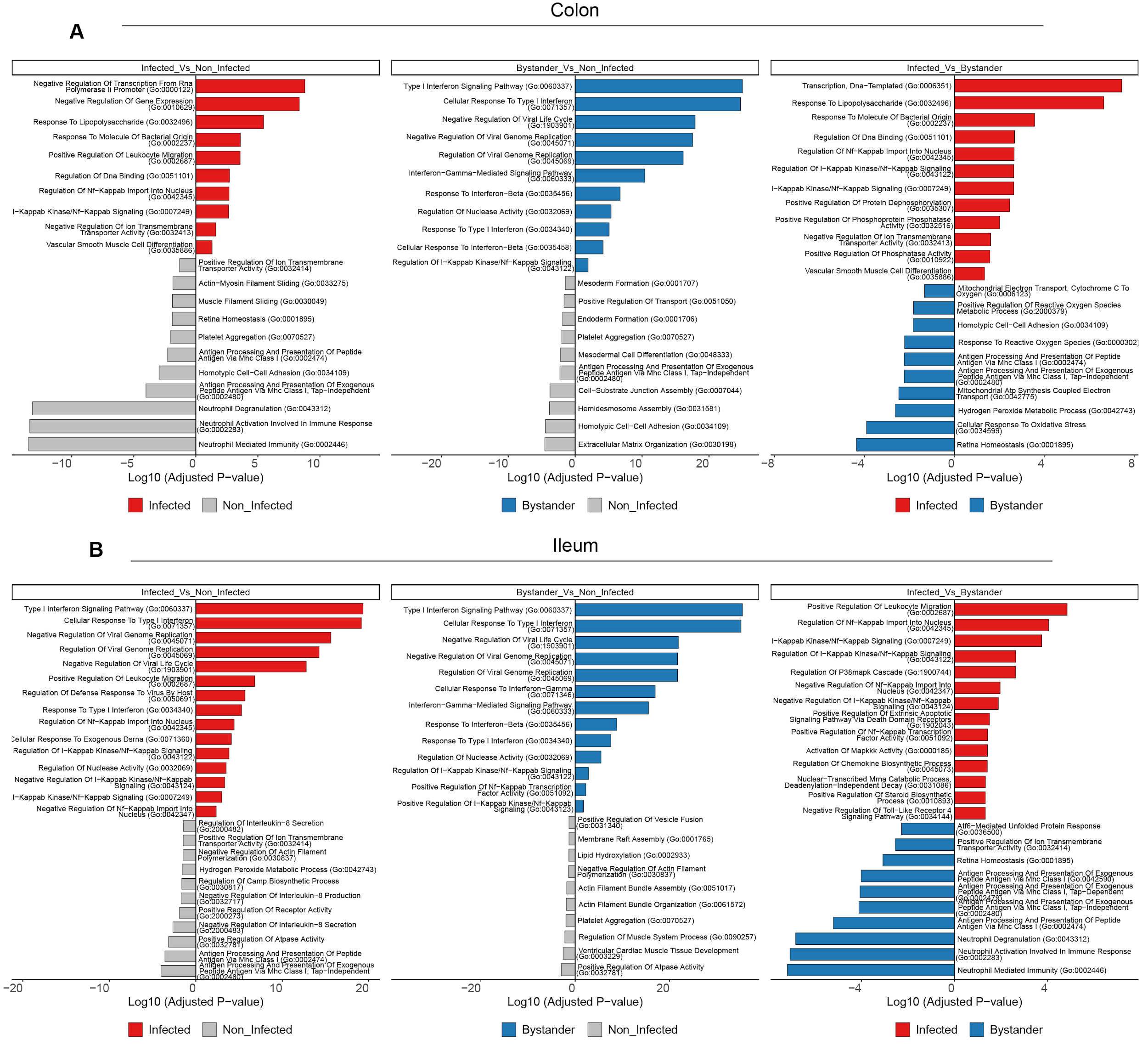
**

**Appendix Figure S4.** **Gene enrichment analysis in infected and bystander cells upon SARS-CoV-2 infection. A.** Gene Ontology (GO) enrichment analysis was performed on the genes that are differentially expressed (FDR<0.05) upon SARS-CoV-2 infection of colon organoids in infected relative to mock cells (left panel), in bystander relative to mock cells (middle panel) and in infected relative to bystander cells (right panel). **B.** same as A. but for ileum organoids

**Appendix Figure S5. Cell type specific differential response of infected *vs.* bystander cells upon SARS-CoV-2 infection of colon-derived organoids.** (top two panels) Volcano plots displaying the genes that are differentially expressed in infected and bystander cells relative to mock-infected cells. The statistical significance (-log10 adjusted p-value) is shown as a function of the log2 fold change. (upper middle panels) Gene Ontology (GO) enrichment analysis was performed on the genes that are differentially expressed (FDR<0.05) upon SARS-CoV-2 infection of colon organoids in infected relative to mock-infected cells (left panel) and in bystander relative to mock-infected cells (right panel). (lower middle panels, left) Dot plot of the top most differentially expressed genes upon SARS-CoV-2 infection in mock, infected and bystander cells at 12 and 24 hpi. The dot size represents the percentage of cells expressing the gene; the color represents the average expression across the cell type. (lower middle panels, right) Dot plot of the top most differentially expressed ISGs upon SARS-CoV-2 infection in mock, infected and bystander cells at 12 and 24 hpi. The dot size represents the percentage of cells expressing the gene; the color represents the average expression across the cell type. (lower panel) Heatmap of signaling pathway enrichment inferred by PROGENy for the given cell type. **A.** Stem cells, **B**. Enterocyte 2. **C**. TA cells, **D.** Cycling TA, **E.** Secretory TA, F. Immature enterocyte 1 and **G.** Enterocyte 1.

**Appendix Figure S6. Cell type specific differential response of infected vs. bystander cells upon SARS-CoV-2 infection of colon-derived organoids.** Same as figure S9 but for **A**. Secretory TA, **B**. Immature enterocyte 1 and C. Enterocyte 1.

**Appendix Figure S7. Cell type specific differential response of infected *vs.* bystander cells upon SARS-CoV-2 infection of ileum-derived organoids.** Same as figure S8 but for ileum organoids. **A.** Enterocyte 1, **B.** TA cells, **C.** Cycling TA, **D.** Stem cells

**
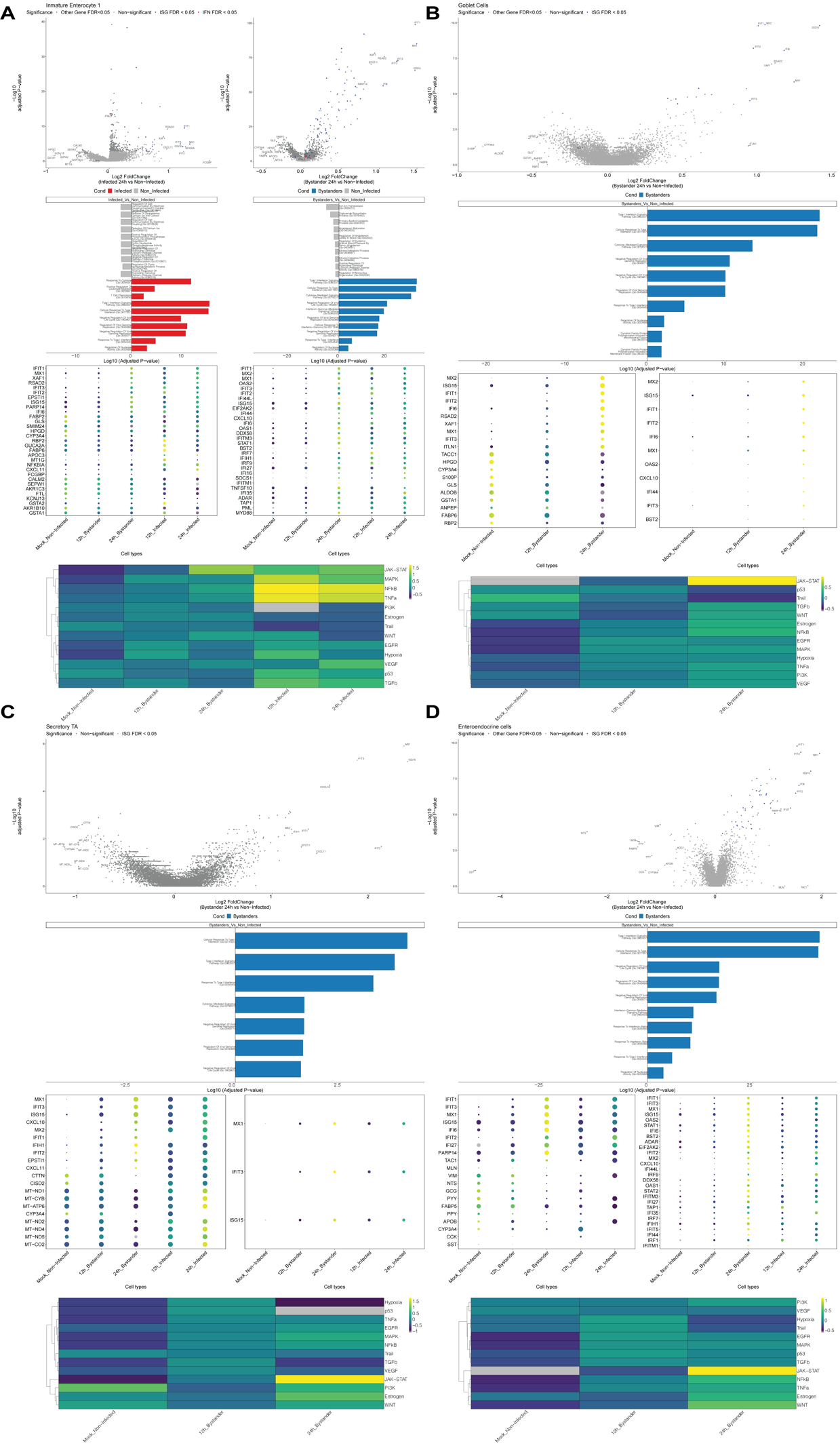
**

**Appendix Figure S8. Cell type specific differential response of infected vs. bystander cells upon SARS-CoV-2 infection of ileum-derived organoids**. Same as figure S11 but for ileum organoids. **A**. Immature enterocyte 1, **B**. Goblet cells and C. Secretory TA.

**Appendix Figure S9. Interferon induction requires virus replication.** Ileum- and colon-derived organoids were seeded in 2D and were infected with live SARS-CoV-2 (-UV) or UV-inactivated SARS-CoV-2 (+UV). At 4, 8, 12 and 24 hpi, RNA was harvested and analyzed for **A-B**. replication of SARS-CoV-2 genome, **C-D**. induction of type III interferon (*IFNλ2/3*) and **E-F.** induction of the interferon-stimulated gene *IFIT1*. **A-F.** N=3 biological replicates were performed for each experiment. Error bars indicate standard deviation.
