# Supplementary material for: Single‐cell analyses reveal SARS‐CoV‐2 interference with intrinsic immune response in the human gut
Source: Mol Syst Biol. 2021 Apr 27;17(4):e10232. doi: 10.15252/msb.202110232 (PMC8077299; doi:10.15252/msb.202110232)
Supplement: Supplementary file 2 — Expanded View Figures PDF [file MSB-17-e10232-s003.pdf]

Expanded View Figures

Figure EV1. Ileum- and colon-derived organoids support SARS-CoV-2 replication and spread Ileum- and colon-derived organoids were seeded in 2D.

A 48 h post-seeding differentiation media was added to cells and the change in differentiation was monitored over indicated time points using q-RT–PCR for markers of stem cells, Paneth cells, Goblet cells, and enterocytes. (*N* = 3 biological replicates).

B 2D seeded organoid cultures were infected with SARS-CoV-2, and the amount of cells was monitored over time by microscopy. (*N* = 3).

C–I 2D seeded organoid cultures were infected with SARS-CoV-2, and infection was assayed. (C) At 4, 8, 12, 16, and 24 hpi, samples were fixed and analyzed by immunofluorescence for the SARS-CoV-2 N protein (red) and dsRNA (green) and nuclei were stained with DAPI (blue). *N* = 3 biological replicates of the same donor were performed, representative images are shown. Scale bar=100 um. (D) Quantification of C. *N* = 3 biological replicates. (E) At indicated times, RNA was harvested, and the amount of virus replication was monitored by q-RT–PCR. *N* = 3 biological replicates of the same donor were performed. (F). At indicated time points, supernatants were collected, and the amounts of de novo infectious viruses produced were titrated on naïve Vero cells. *N* = 4 biological replicates were performed. (G-I). At indicated time points, RNA was harvested and the upregulation of IFNβ1, INFλ1, and IFNλ2/3 was analyzed by q-RT–PCR. *N* = 3 biological replicates of the same donor were performed.

Data information: (A-F) Error bar indicates standard deviation. *P* was determined by unpaired *t*-test.

▶

 © 2021 The Authors

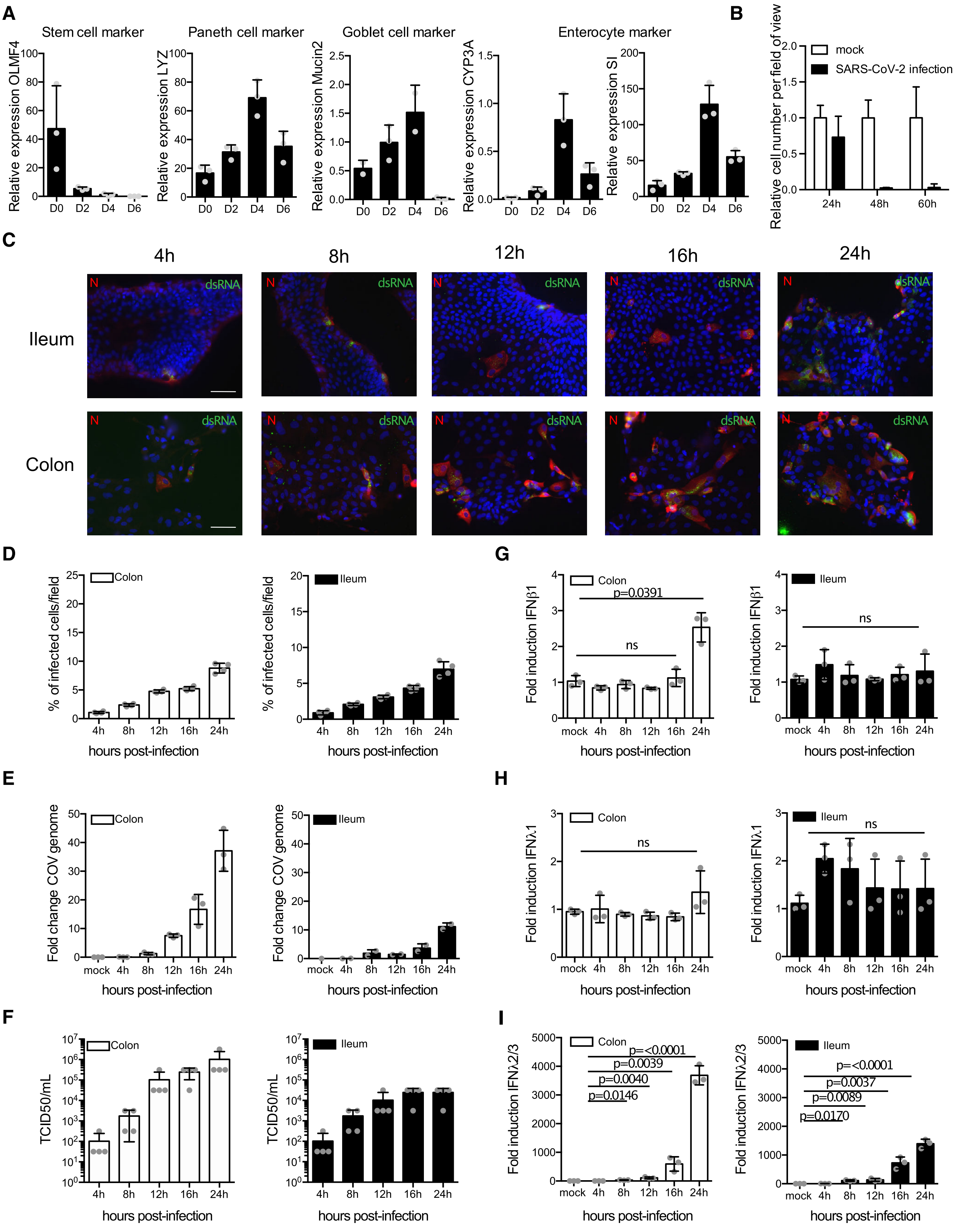
A
Stem cell marker
Paneth cell marker
Goblet cell marker
Enterocyte marker
Relative expression OLMF4
Relative expression LYZ
Relative expression Mucin2
Relative expression CYP3A
Relative expression SI
D0 D2 D4 D6
B
mock
SARS-CoV-2 infection
Relative cell number per field of view
24h 48h 60h
C
4h 8h 12h 16h 24h
Ileum
Colon
N
dsRNA
D
Colon
Ileum
% of infected cells/field
hours post-infection
E
Fold change COV genome
mock 4h 8h 12h 16h 24h
F
TCID50/mL
G
Fold induction IFNβ1
p=0.0391
ns
H
Fold induction IFNλ1
I
Fold induction IFNλ2/3
p=<0.0001
p=0.0039
p=0.0040
p=0.0146
p=0.0037
p=0.0089
p=0.0170

Figure EV1.

Figure EV2. Clustering and annotation of colon and ileum organoids.

A Uniform manifold approximation and projection (UMAP) embedding of single-cell RNA-Seq data from human ileum organoids colored by the unsupervised clusters.
B Heatmap of relative expression of the top three marker genes for each cluster.
C UMAP colored by cell type.
D Heatmap showing Pearson correlations between the average expression of every gene across cell types between infected and mock samples.
E Mapping between organoids cell types and transfer label from tissue cell types.
F–J Same as (A–E) but for ileum organoids.

 © 2021 The Authors

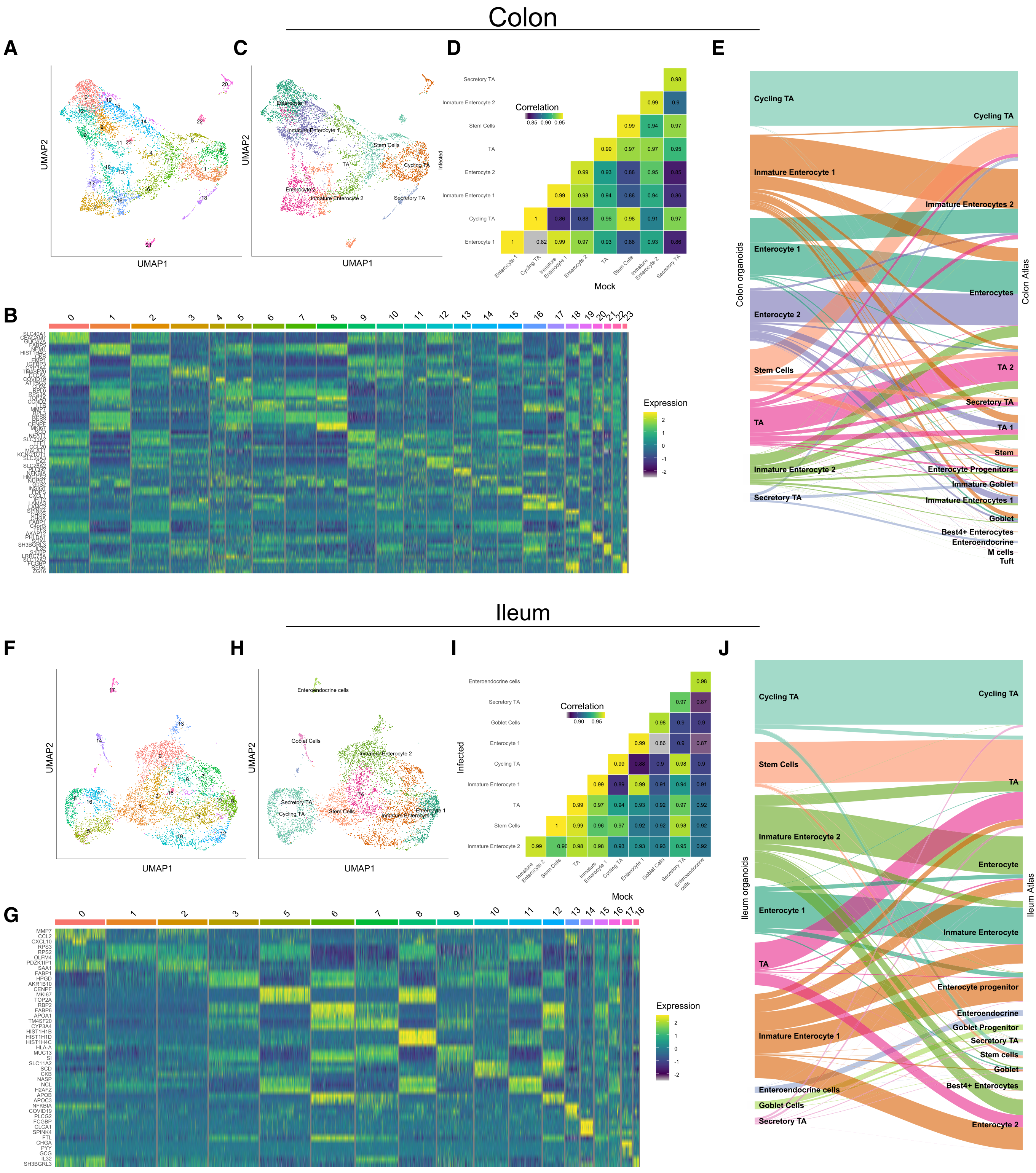
Colon
A
C
D
E
B
Ileum
F
H
I
J
G
UMAP1
UMAP2
Correlation
Expression
Mock
Infected
Colon organoids
Colon Atlas
Ileum organoids
Ileum Atlas

Figure EV2.

Figure EV3. Identification of SARS-CoV-2 infected cells using targeted scRNAseq datasets.

A, B Proportion of cells infected with SARS-CoV-2 for each sample and violin plots displaying SARS-CoV-2 expression for the 10x genomics 3' scRNAseq and for the targeted scRNAseq for mock-infected and SARS-CoV-2-infected colon and ileum organoid at 12 and 24 hpi.

C (Top panels) SARS-CoV-2 expression as function of the number of genes per droplet from the targeted scRNAseq datasets of colon organoids at 12 and 24 hpi. Droplet types are colored for droplets containing cells and empty droplets. The vertical line represents the threshold for droplets containing $<$ 2,000 genes per cell. The horizontal line represents the threshold used to define the baseline of infection at 12 and 24 hpi. (Bottom panels) Uniform manifold approximation and projection (UMAP) embedding of the scRNAseq data of infected colon organoids depicting SARS-CoV-2 infected cells. (Left) Relative expression of SARS-CoV-2 in non-corrected datasets. (Right) Relative expression of SARS-CoV-2 in corrected datasets using the thresholds determined in top panel dotplots.

D Same as (C) but for ileum organoids.

E, F (Top panels) Correlation of the expression of each gene in 10x genomics 3' scRNAseq against their expression in targeted scRNAseq across conditions. (Bottom panels) SARS-CoV-2 expression in each infected cell in 10x genomics 3' scRNAseq against targeted scRNAseq. Colon (E) and Ileum (F).

 © 2021 The Authors

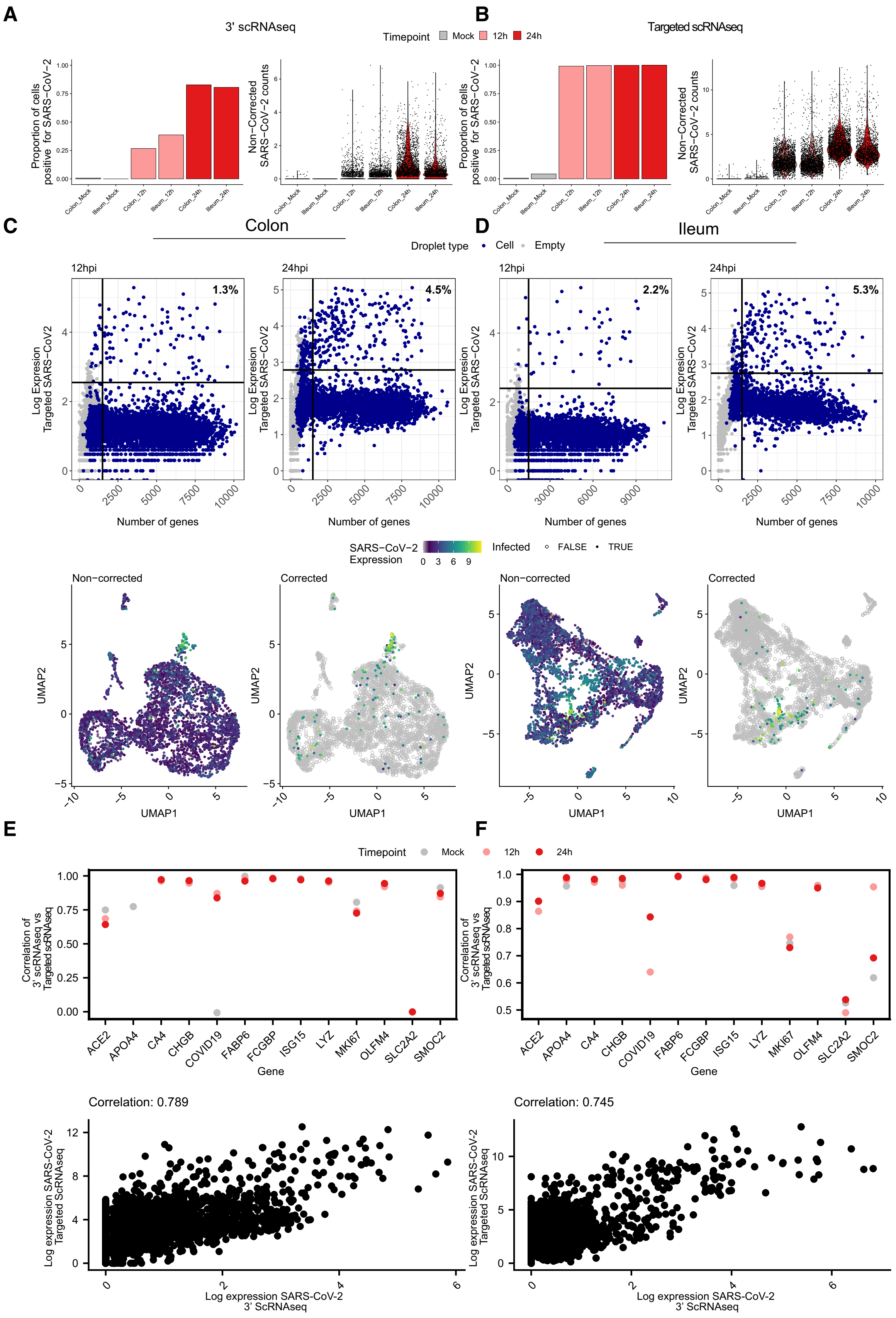
A
3' scRNAseq
B
Targeted scRNAseq
Timepoint Mock 12h 24h
Proportion of cells positive for SARS−CoV−2
Non−Corrected SARS−CoV−2 counts
Colon_Mock
Ileum_Mock
Colon_12h
Ileum_12h
Colon_24h
Ileum_24h
C
Colon
D
Ileum
Droplet type Cell Empty
12hpi
24hpi
1.3%
4.5%
2.2%
5.3%
Log Expression Targeted SARS−CoV2
Number of genes
SARS−CoV−2 Expression
Infected FALSE TRUE
Non−corrected
Corrected
UMAP1
UMAP2
E
F
Correlation of 3' scRNAseq vs Targeted scRNAseq
ACE2
APOA4
CA4
CHGB
COVID19
FABP6
FCGBP
ISG15
LYZ
MKI67
OLFM4
SLC2A2
SMOC2
Gene
Correlation: 0.789
Correlation: 0.745
Log expression SARS-CoV-2 Targeted ScRNAseq
Log expression SARS-CoV-2 3' ScRNAseq

Figure EV3.

Figure EV4. Expression of SARS-CoV-2 and ACE2 in colon- and ileum-derived organoids.

A Uniform manifold approximation and projection (UMAP) embedding of the scRNAseq data of infected colon organoids at 12 and 24 hpi, colored by the corrected targeted normalized expression of SAR-Cov-2. Colon (left) and Ileum (right).

B Same as (A) but for ACE2 and TMPRSS2 relative expression for mock-infected.

C Total transcript counts in each cell type for mock-infected and SARS-CoV-2-infected and bystanders cells in colon and ileum organoids at 12 hpi and 24 hpi. The boxes represent the interquartile range, the horizontal line in the box is the median, and the whiskers represent 1.5 times the interquartile range (Colon; Enterocyte 1 Mock Non-Infected *n* = 326, 12 h Bystander *n* = 481, 24 h Infected *n* = 4 & 24 h Bystander *n* = 375, Cycling TA Mock Non-Infected *n* = 273, 12 h Infected *n* = 3, 12 h Bystander *n* = 361, 24 h Infected *n* = 6 & 24 h Bystander *n* = 420, Immature Enterocyte 1 Mock Non-Infected *n* = 414, 12 h Infected *n* = 2, 12 h Bystander *n* = 571, 24 h Infected *n* = 7 & 24 h Bystander *n* = 448, Enterocyte 2 Mock Non-Infected *n* = 402, 12 h Infected *n* = 4, 12 h Bystander *n* = 350, 24 h Infected *n* = 11 & 24 h Bystander *n* = 245, TA Mock Non-Infected *n* = 244, 12 h Infected *n* = 7, 12 h Bystander *n* = 324, 24 h Infected *n* = 21 & 24 h Bystander *n* = 293, Stem Cells Mock Non-Infected *n* = 268, 12 h Infected *n* = 1, 12 h Bystander *n* = 324, 24 h Infected *n* = 3 & 24 h Bystander *n* = 207, Immature Enterocyte 2 Mock Non-Infected *n* = 208, 12 h Infected *n* = 15, 12 h Bystander *n* = 152, 24 h Infected *n* = 47 & 24 h Bystander *n* = 169, Secretory TA Mock Non-Infected *n* = 36, 12 h Infected *n* = 2, 12 h Bystander *n* = 53, 24 h Infected *n* = 4 & 24 h Bystander *n* = 67. Ileum; Immature Enterocyte 2 Mock_Non-Infected *n* = 459, 12 h_Infected *n* = 31, 12 h_Bystander *n* = 476, 24 h_Infected *n* = 50 & 24 h_Bystander *n* = 332, Stem Cells Mock_Non-Infected *n* = 248, 12 h_Infected *n* = 2, 12 h_Bystander *n* = 310, 24 h_Infected *n* = 1 & 24 h_Bystander *n* = 166, TA Mock_Non-Infected *n* = 272, 12 h_Infected *n* = 5, 12 h_Bystander *n* = 269, 24 h_Infected *n* = 5 & 24 h_Bystander *n* = 144, Immature Enterocyte 1 Mock_Non-Infected *n* = 465, 12 h_Infected *n* = 2, 12 h_Bystander *n* = 616, 24 h_Infected *n* = 8 & 24 h_Bystander *n* = 282, Cycling TA Mock_Non-Infected *n* = 320, 12 h_Infected *n* = 8, 12 h_Bystander *n* = 464,24 h_Infected *n* = 15 & 24 h_Bystander *n* = 393, Enterocyte 1 Mock_Non-Infected *n* = 253, 12 h_Infected *n* = 9, 12 h_Bystander *n* = 370, 24 h_Infected *n* = 4 & 24 h_Bystander *n* = 138, Goblet Cells Mock_Non-Infected *n* = 36, 12 h_Bystander *n* = 60 & 24 h_Bystander *n* = 31, Secretory TA Mock_Non-Infected *n* = 19, 12 h_Infected *n* = 1, 12 h_Bystander *n* = 80, 24 h_Infected *n* = 1 & 24 h_Bystander *n* = 10, Enteroendocrine cells Mock_Non-Infected *n* = 35, 12 h_Infected *n* = 3, 12 h_Bystander *n* = 38, 24 h_Infected *n* = 1 & 24 h_Bystander *n* = 26).

© 2021 The Authors

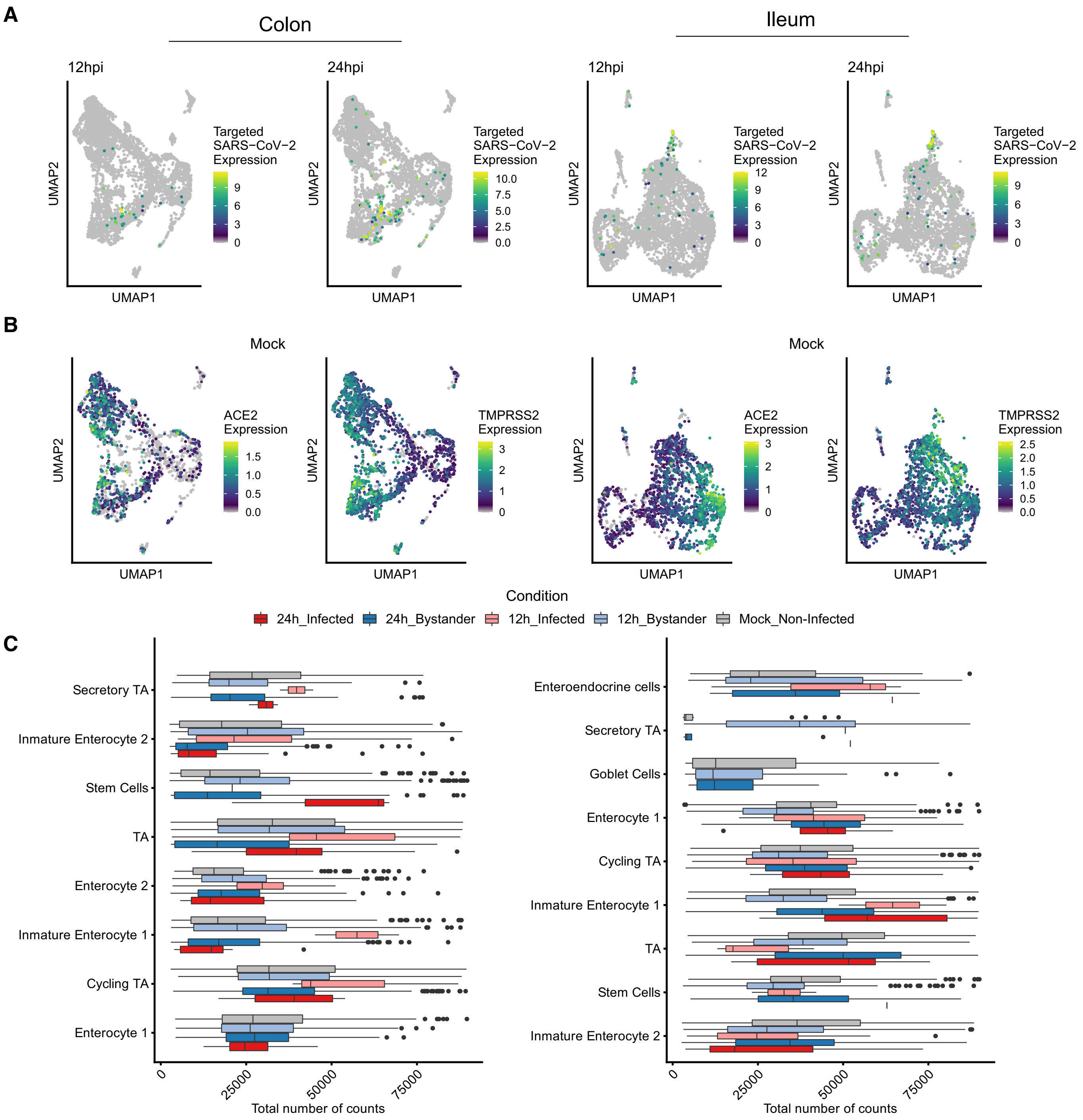
A
Colon
Ileum
12hpi
24hpi
Targeted SARS−CoV−2 Expression
UMAP1
UMAP2
B
Mock
ACE2 Expression
TMPRSS2 Expression
C
Condition
24h_Infected
24h_Bystander
12h_Infected
12h_Bystander
Mock_Non-Infected
Secretory TA
Inmature Enterocyte 2
Stem Cells
TA
Enterocyte 2
Inmature Enterocyte 1
Cycling TA
Enterocyte 1
Enteroendocrine cells
Goblet Cells
Total number of counts

Figure EV4.

Figure EV5. Differential response of infected and bystander cells to SARS-CoV-2 infection.

A–F Volcano plots displaying the genes that are differentially expressed upon SARS-CoV-2 infection of colon organoids. (A, B) infected vs. mock-infected cells at 12 hpi (A) and 24 hpi (B), (C, D) bystander vs. mock-infected cells at 12 hpi (C) and 24 hpi (D), and (E, F) infected vs. bystander cells at 12 hpi (E) and 24 hpi (F). The statistical significance (-log10 adjusted *P*-value) is shown as a function of the log2 fold change. MAST tests were used to generate *P*-values, bonferroni multiple hypotheses correction was used to compute *FDR values*. Labeled dots in blue in all panels are gene names of selected differentially expressed genes between the compared two populations. Labeled dots in red in all panels are gene corresponding to interferon if detected.

G–L Same as (A–F) but for ileum organoids.

 © 2021 The Authors

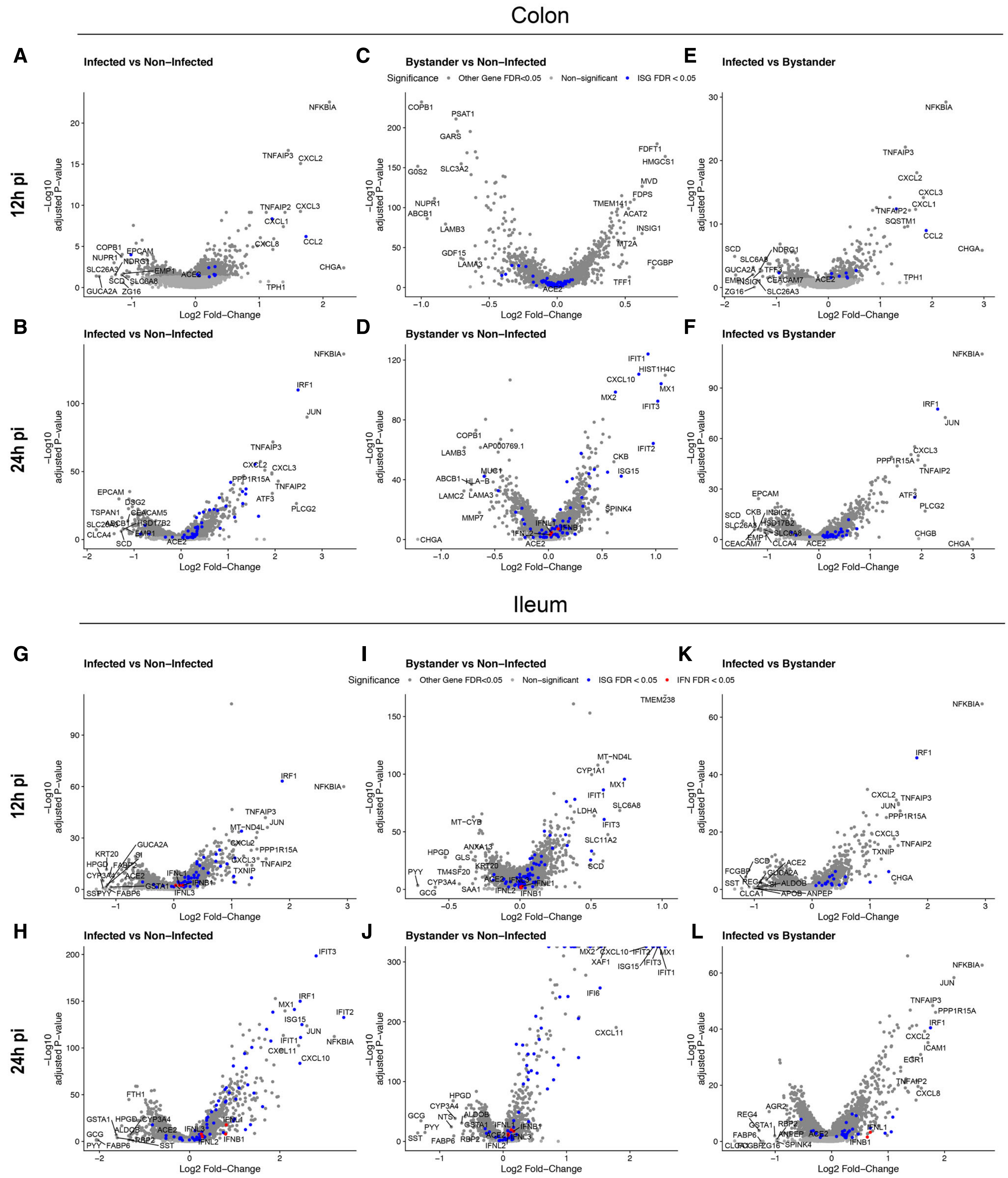
Colon
A Infected vs Non–Infected
B Infected vs Non–Infected
C Bystander vs Non–Infected
D Bystander vs Non–Infected
E Infected vs Bystander
F Infected vs Bystander
Significance • Other Gene FDR<0.05 • Non–significant • ISG FDR < 0.05
12h pi
24h pi
Ileum
G Infected vs Non–Infected
H Infected vs Non–Infected
I Bystander vs Non–Infected
J Bystander vs Non–Infected
K Infected vs Bystander
L Infected vs Bystander
Significance • Other Gene FDR<0.05 • Non–significant • ISG FDR < 0.05 • IFN FDR < 0.05
−Log10 adjusted P–value
Log2 Fold–Change

Figure EV5.
